# The Inhibitory Activity of Luzonicosides from the Starfish *Echinaster luzonicus* against Human Melanoma Cells

**DOI:** 10.3390/md15070227

**Published:** 2017-07-18

**Authors:** Olesya S. Malyarenko, Sergey A. Dyshlovoy, Alla A. Kicha, Natalia V. Ivanchina, Timofey V. Malyarenko, Bokemeyer Carsten, von Amsberg Gunhild, Valentin A. Stonik, Svetlana P. Ermakova

**Affiliations:** 1 Laboratory of Enzyme Chemistry, G.B. Elyakov Pacific Institute of Bioorganic Chemistry, Far-Eastern Branch, Russian Academy of Sciences, Vladivostok 690022, Russia; malyarenko.os@gmail.com; 2 Laboratory of Marine Natural Products Chemistry, G.B. Elyakov Pacific Institute of Bioorganic Chemistry, Far-Eastern Branch, Russian Academy of Sciences, Vladivostok 690022, Russia; dyshlovoy@gmail.com (S.A.D.); kicha@piboc.dvo.ru (A.A.K.); ivanchina@piboc.dvo.ru (N.V.I.); malyarenko-tv@mail.ru (T.V.M.); stonik@piboc.dvo.ru (V.A.S.); 3 Department of Oncology, Hematology and Bone Marrow Transplantation with Section Pneumology, Hubertus Wald-Tumorzentrum, University Medical Center Hamburg-Eppendorf, Hamburg 20246, Germany; c.bokemeyer@uke.de (B.C.); g.von-amsberg@uke.de (v.A.G.); 4 School of Natural Sciences, Far East Federal University, Vladivostok 690922, Russia

**Keywords:** starfish, *Echinaster luzonicus*, steroids, cyclic glycosides, melanoma, cytotoxicity, cell cycle, apoptosis, migration

## Abstract

Malignant melanoma is the most dangerous form of skin cancer, with a rapidly increasing incidence rate. Despite recent advances in melanoma research following the approval of several novel targeted and immuno-therapies, the majority of oncological patients will ultimately perish from the disease. Thus, new effective drugs are still required. Starfish steroid glycosides possess different biological activities, including antitumor activity. The current study focused on the determination of the in vitro inhibitory activity and the mechanism of action of cyclic steroid glycosides isolated from the starfish *Echinaster luzonicus*—luzonicoside A (LuzA) and luzonicoside D (LuzD)—in human melanoma RPMI-7951 and SK-Mel-28 cell lines. LuzA inhibited proliferation, the formation of colonies, and the migration of SK-Mel-28 cells significantly more than LuzD. Anti-cancer activity has been ascribed to cell cycle regulation and apoptosis induction. The molecular mechanism of action appears to be related to the regulation of the activity of cleaved caspase-3 and poly(ADP-ribose) polymerase (PARP), along with Survivin, Bcl-2, p21 and cyclin D1 level. Overall, our findings support a potential anti-cancer efficacy of luzonicosides A and D on human melanoma cells.

## 1. Introduction

All over the world, the incidence of malignant melanoma is higher than other cancers. Despite the rapid progress in the diagnosis and treatment of different types of cancer, the prognosis for metastatic melanoma remains very limited. In recent years, natural compounds, especially of marine origin, have proved to have great potential in the prevention and therapy of serious diseases, including cancer [1].

Starfish are a notably rich source of structurally diverse polar steroid glycosides. On the basis of their chemical structures, starfish steroid glycosides may be subdivided into mono and biglycosides (rarely, triglycosides) of polyhydroxysteroids, oligoglycosides (known as asterosaponins), and rare glycosides with cyclic carbohydrate chains. Starfish steroid glycosides were found to have cytotoxic, neuritogenic, hemolytic, antiviral, antibacterial, antitumor, antifouling, antifungal, and cancer-preventive effects [2,3,4,5]. In particular, several asterosaponins and steroid mono or biglycosides possess inhibitory activities against different cancer cell lines [6]. For example, novaeguinoside II and asterosaponin 1 from the starfish *Culcita novaeguineae* have cytostatic and pro-apoptotic activities against human glioblastoma U87MG cells [7]. Furthermore, asterosaponin 1 was shown to induce apoptosis through the regulation of endoplasmic reticulum (ER-apoptosis) and, consequently, decreased the proliferation rate of human lung cancer A549 cells [8]. The anti-proliferative effect of polyhydroxysteroid glycosides from the starfish *Anthenea chinensis* has been associated with the prevention of tubulin polymerization-promotion in human glioblastoma cells [9]. Archasteroside B from the starfish *Archaster typicus* activated basal AP-1 and p53 transcriptional factors, but had no effect on NF-κB factors in mouse epidermal JB6 Cl41 cells [10]. Asterosaponins from the starfish *Astropecten monacanthus* possessed anti-proliferative and pro-apoptotic activities in human promyelocytic leukemia cells HL-60, prostate cancer cells PC-3, and gastric cancer cells SNU-C5, and regulated the activity of mitogen-activated protein kinases (MAPKs) and phosphatidyl inositol 3 (PI3K)/AKT kinases [11]. Polar steroid glycosides, isolated from the starfish *Hippasteria kurilensis, Asteropsis carinifera, Lethasterias fusca*, and *Lethasterias ochotensis,* effectively inhibited the formation and growth of colonies of human melanoma, breast adenocarcinoma, and colorectal carcinoma cells [12,13,14,15]. Taken together, these findings revealed that polar steroid glycosides from the starfishes might be promising candidates for the prevention and/or therapy of different malignancies, and intensive investigations of their antitumor properties and molecular mechanisms of action are needed.

**Figure 1:**
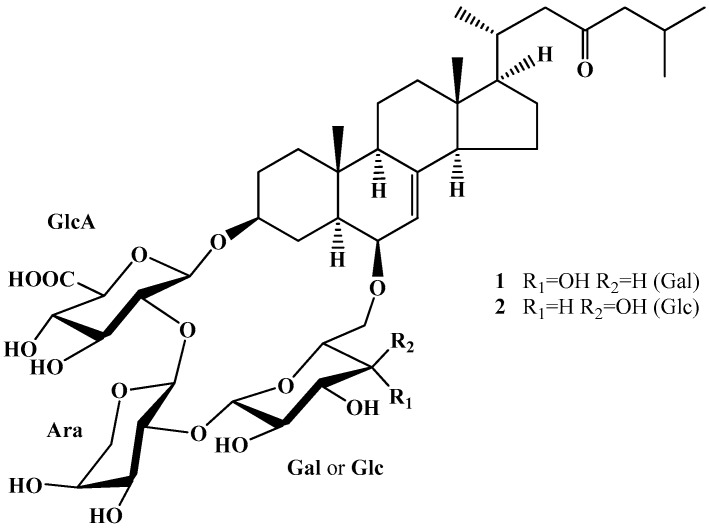
Structures of luzonicoside A (LuzA) (1) and luzonicoside D (LuzD) (2) isolated from the starfish *E. luzonicus.*

A unique group of starfish steroid glycosides are the cyclic glycosides. These glycosides differ from other common starfish steroid glycosides in several structural peculiarities, such as a trisaccharide chain, which forms a macrocycle between C-3 and C-6 of aglycone moiety; Δ^7^-3β,6β-dihydroxysteroid aglycone; and the presence of a glucuronic acid residue in the carbohydrate moiety. Glycosides with cyclic carbohydrate chains have so far been found only in two species of the genus *Echinaster* and in one species of the genus *Leiaster* [16,17,18]. To date, only nine representatives of this structural group have been reported. Recently, the structures and in vitro immunomodulatory activity of two cyclic steroid glycosides from the starfish *Echinaster luzonicus—*characterized earlier as luzonicoside A (LuzA) and the newer luzonicoside D (LuzD) (Figure 1)—were studied [16].

Here, we report the in vitro inhibitory activity and potential molecular mechanism of action of LuzA and LuzD in human malignant melanoma cells, with a specific focus on cytotoxicity, cancer cell proliferation, the cell cycle, apoptosis, colony formation, and cancer cell migration.

## 2. Results and Discussion

### 2.1. Cytotoxicity and Inhibition of Malignant Melanoma Cell Proliferation by Luzonicosides A and D

**Figure 2:**
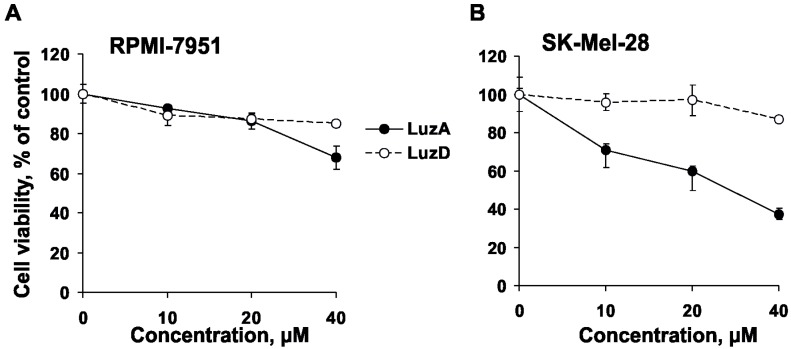
The effect of LuzA and LuzD on human melanoma cell proliferation. (**A**) Human melanoma cells RPMI-7951 (1 × 10^4^ cells/well) and (**B**) SK-Mel-28 (1 × 10^4^ cells/well) were seeded in 96-well plates in 200 µL of Dulbecco’s Modified Eagle’s medium (DMEM), then treated with LuzA and LuzD (10, 20, and 40 µM), or their vehicle—DMSO—as a negative control, for 72 h. Cell proliferation was determined by MTS assay. Data are represented as the means ± standard deviation (SD) as determined from triplicate experiments.

The cytotoxic effect of LuzA and LuzD (Figure 1) (up to 200 µM) against human malignant melanoma cells RPMI-7951 and SK-Mel-28 was evaluated by CellTiter 96^®^ AQueous One Solution (MTS) assay. LuzA and LuzD did not show cytotoxic activity against RPMI-7951 cells at concentrations up to 200 µM, while an IC_50_ of 119 and of 160 µM was determined for SK-Mel-28 cells, respectively (data not shown). Therefore, we chose the non-cytotoxic concentrations of 10, 20, and 40 µM of LuzA and LuzD for subsequent experiments. In the next step, the anti-proliferative activity of the compounds was examined. LuzA decreased the proliferation of RPMI-7951 cells by 8%, 14%, and 32% after 3 days of treatment (Figure 2A), and of SK-Mel-28 cells by 29%, 40%, and 63% at concentrations of 10, 20, and 40 µM, respectively (Figure 2B). LuzD exerted moderate anti-proliferative activity against tested cell lines, with an inhibition of less than 15% (Figure 2A,B). Therefore, LuzA showed a more pronounced anti-proliferative effect in human malignant melanoma cells than LuzD. The results are correlated with the previously obtained data, which described a slight cytotoxic activity of polar steroids from the starfishes against human malignant melanoma RPMI-7951 cells [15,19].

### 2.2. The Cell Cycle Regulation and Induction of Apoptosis of Malignant Melanoma Cells by Luzonicosides A and D

Cell cycle progression is one of the basic and most significant cancer-related processes. The cell cycle includes phases of cell growth (interphase) and cell division (M phase). The interphase consists of several steps: the S phase (the replication of DNA), the G1 phase (in which the cell increases in size), and the G2 phase (the preparation for cell division). Based on the stimulatory and inhibitory signals, the cell receives, and it “decides” whether or not it should enter the cell cycle and divide [20]. In non-malignant cells, the cell cycle is regulated by several signaling pathways responsible for cell growth, DNA replication and cell division. However, in cancer patients, genetic mutations lead to uncontrolled cell division and a malfunction in the apoptosis process. Apoptosis is a sequence of complex biochemical events involving different signal pathways, finally resulting in programmed cell-death under physiological and pathological conditions. The mutations can occur at any point along these signaling cascades, which lead to neoplastic cell transformation, metastasis, and chemo-resistance [21]. Consequently, the search for, and investigation of, compounds which are able to regulate the cell cycle and induce cancer cell-specific apoptosis is one of the principal directions in cancer therapy.

**Figure 3:**
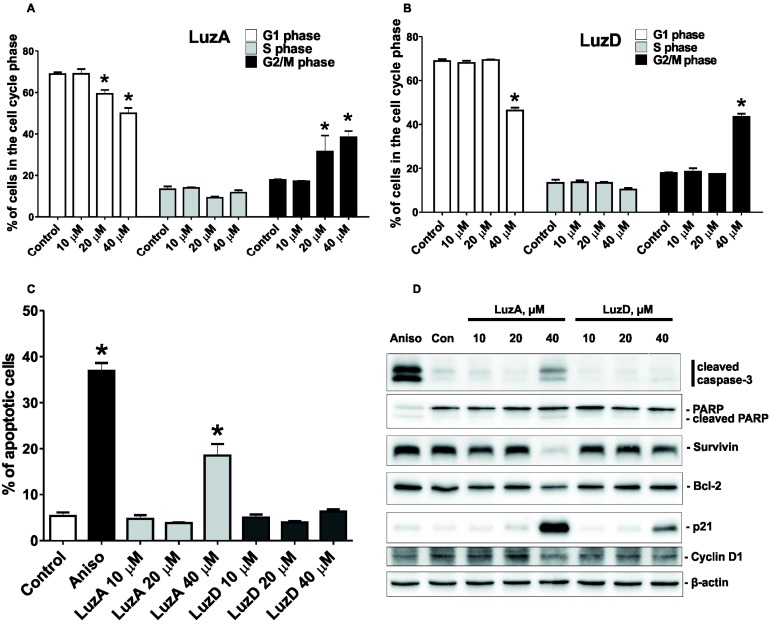
The effect of LuzA and LuzD on cell cycle regulation and the apoptosis induction of human melanoma cells. SK-Mel-28 cells (2 × 10^5^ cells/well) were treated with LuzA (**A**) and LuzD (**B**) at doses of 10, 20, and 40 µM for 48 h. Then, cells were harvested with a trypsin-ethylenediaminetetraacetic acid (EDTA) solution, fixed with 70% EtOH/H_2_O, stained with PI/RNase buffer, and analysed by fluorescence-activated cell sorting (FACS). The results were quantitatively analyzed by Cell Quest Pro software (BD Bioscience, San Jose, CA, USA). The amount of apoptotic cells was detected as a sub-G1 population containing different concentrations of LuzA and LuzD. After 48 h of incubation, cells were harvested with a trypsin-EDTA solution, fixed with 70% EtOH/H_2_O, stained with PI/RNase buffer, and analysed by FACS. The results were quantitatively analyzed using the Cell Quest Pro software (BD Bioscience, San Jose, CA, USA). The asterisk (*) indicates a significant increasing of the amount of cells in the cell cycle phase treated with glycosides compared with the non-treated cells (* *p* < 0.05). (**C**) The amount of the apoptotic cells was detected as a sub-G1 population. The asterisk (*) indicates a significant increasing of the amount of apoptotic cells treated with glycosides compared with the non-treated cells (* *p* < 0.05). (**D**) The activation of cleaved caspase-3, PARP, cleaved PARP, Survivin, p21, Bcl-2, and Cyclin D1. SK-Mel-28 cells were treated with 10, 20, and 40 µM of LuzA and LuzD and incubated for 48 h. After drug exposure, total protein lysates were prepared. The protein samples (30 µg) were subjected to sodium dodecyl sulfate polyacrylamide gel electrophoresis (SDS-PAGE) and followed by detection with immunoblotting, using antibodies against cleaved caspase-3 (19 kDa), PARP (116 kDa), cleaved PARP (89 kDa), Survivin (16.5 kDa), p21 (21 kDa), Bcl-2 (28 kDa), and Cyclin D1 (36 kDa) proteins.

To investigate the effect of LuzA and LuzD (10, 20, and 40 µM) on cell cycle progression and apoptosis induction, we chose SK-Mel-28 cells, because LuzA and LuzD possessed more prominent anti-proliferative activity against this cell line than against RPMI-7951 cells (Figure 2A,B). Both LuzA and LuzD were able to induce G2/M cell cycle arrest in cells treated for 48 h (Figure 3A,B). LuzA was found to increase the fraction of apoptotic cells by 18.5% at a concentration of 40 µM (Figure 3C). In addition, LuzD slightly induced the apoptosis of SK-Mel-28 cells at the same dosage (Figure 3C). These observations were in accordance with the results of the cytotoxicity and proliferation assays.

### 2.3. The Molecular Mechanism of the Pro-Apoptotic Effect of Luzonicosides A and D

In order to investigate the molecular mechanisms underlying the observed effects of LuzA and LuzD, we analyzed several apoptosis- and cell cycle-related proteins (Figure 3D).

There are several pathways for apoptosis induction. The intrinsic pathways (or mitochondrial) and extrinsic (or death receptor) pathways are usually described; both pathways eventually lead to the execution phase of apoptosis. The number of investigations devoted to the intrinsic endoplasmic reticulum pathway of apoptosis induction is limited [22]. The mitochondrial pathway is known to be induced by different factors [23]. Pro- and anti-apoptotic molecules participate in the realization of mitochondrial apoptosis. The Bcl-2 family is represented by the anti-apoptosis proteins Bcl-2 and Bcl-xL, as well as the pro-apoptosis proteins Bax, Bid, and Bak. The pro-apoptotic members of the Bcl-2 family induce apoptosis via the release of cytochrome C and the activation of caspase-9, which activates ‘initiator’ caspases such as caspase-3. Activated caspase-3, in turn, cleaves its specific substrate, poly(ADP-ribose) polymerase (PARP), finally inducing apoptosis [24].

LuzA induced the cleavage of caspase-3 and PARP, as well as decreasing the anti-apoptotic protein Bcl-2 at 40 µM. At the same time, cells treated with 40 µM of LuzD did not possess any significant effect on the expression level of these proteins. Both compounds induced the down-regulation of the anti-apoptotic Survivin. In line with previous experiments, the effect was more pronounced in cells treated with LuzA (Figure 3D).

p21 (also known as p21WAF1/Cip1) is a cyclin-dependent kinase inhibitor which is able to arrest the cell cycle for a variety of reasons. The main functional role of p21 is to bind and down-regulate the activity of the cyclin-dependent kinases (CDKs). This will lead to cell cycle arrest at specific stages and the inhibition of cell growth [25].

In our experiments, the up-regulation of the cell cycle-related p21 and the down-regulation of the expressional levels of Cyclin D1 were observed. These effects were shown to be related to G2/M cell cycle arrest, which is in line with our previous results (Figure 3D).

Recently, several groups have reported the effects of asterosaponins on the apoptosis induction of human cancer cells. For example, Cheng et al. showed that asterosaponin 1 from the starfish *Culcita novaeguineae* significantly suppressed human glioblastoma U87MG cell proliferation by the induction of apoptosis. Its molecular mechanism of anti-proliferative effects was related to the regulation of the expression of anti- and pro-apoptosis proteins (Bcl-2, and Bax) and DNA fragmentation [26]. Additionally, asterosaponin 1 suppressed the proliferation of human lung cancer A549 cells via ER-apoptosis [8]. Later, the same group of authors found that novaeguinoside II (asterosaponin from the starfish *Culcita novaeguineae*) was able to induce apoptosis in U87MG cells by increasing cytochrome C and caspase-3 protein expression levels [7]. Asterosaponin asterioside D from the starfish *Astropecten monacanthus* was found to induce the apoptosis of different types of cancer cells (HL-60, PC-3, and SNU-C5) by decreasing the activity of PI3K/AKT and extracellular signal-regulated kinase (ERK) 1/2 kinases and by the down-regulation of c-myc expression [11].

Our data provide the evidence that LuzA and LuzD from the starfish *Echinaster luzonicus* inhibit the proliferation of human melanoma SK-Mel-28 cells by cell cycle regulation and the induction of apoptosis. The alteration of the expression of p21, cyclin D1, caspase-3, Bcl-2, and Survivin proteins was identified as a potential mechanism of action.

### 2.4. The Inhibition of Malignant Melanoma Cell Colony Formation by Luzonicosides A and D

**Figure 4:**
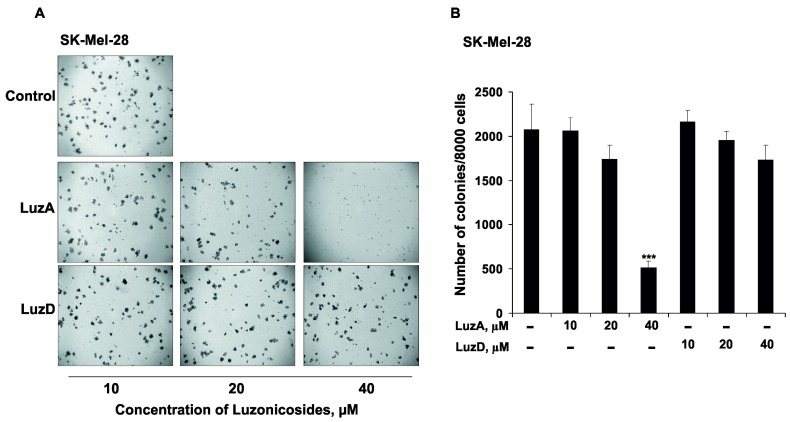
The inhibitory effect of LuzA and LuzD on the colony formation of human malignant melanoma cells. (**A**) SK-Mel-28 cells (2.4 × 10^4^/mL) treated with LuzA and LuzD (10, 20, and 40 µM) were exposed to 1 mL of 0.3% Basal Medium Eagle (BME)’s agar containing 10% fetal bovine serum (FBS). The culture was maintained at 37°C in a 5% CO_2_ atmosphere for 3 weeks. (**B**) The colonies of cells were photographed under a microscope and scored using the ImageJ software program. Data are represented as means ± SD of colony numbers determined in three independent experiments. The asterisk (*) indicates a significant decrease in colony formation in cells treated with luzonicosides compared with the non-treated cells (*** *p* < 0.001).

Colony formation in a soft agar matrix is a commonly used method in cell biology. It allows the detection of the capability of neoplastic cells, which develop separately on a solid surface in vitro, and the determination of the effect of different compounds on this process. It is assumed to be a highly stringent test for malignant cell transformation in vitro [27]. Therefore, we used this assay to evaluate the effect of LuzA and LuzD on the colony formation of SK-Mel-28 cells. LuzA reduced colony numbers of SK-Mel-28 by 16% and 75% at 20 and 40 µM, respectively (Figure 4). LuzD revealed moderate activity, with a suppression of colony formation of only 17% at 40 µM (Figure 4), similar to our previous experiment.

We have recently demonstrated the inhibitory activity of starfish polar steroids on the colony growth of cancer cells [12,13,14,15]. Thus, several asterosaponins and other steroid glycosides from the starfish *Hippasteria kurilensis*, *Asteropsis carinifera, Lethasterias fusca*, and *Leptasterias ochotensis* exhibited a significant suppression of the colony formation of human colorectal carcinoma, melanoma, and breast adenocarcinoma cells in soft agar [12,13,14,15].

### 2.5. The Inhibition of Malignant Melanoma Cell Migration by Luzonicosides A and D

**Figure 5:**
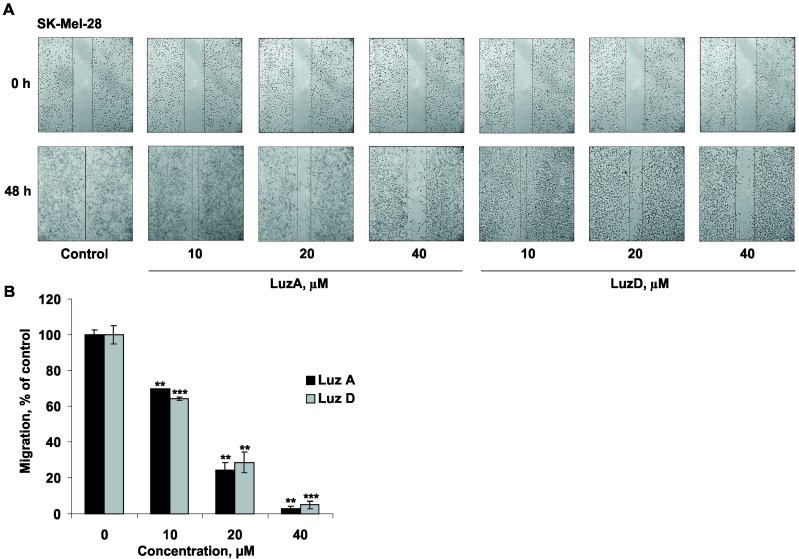
The effect of LuzA and LuzD on the migration of human melanoma cells. (**A**) Cells (3.0 × 10^5^ cells/mL) were seeded in 6-well plates. When cells were grown to an 80% confluence, the medium was removed and a wound was scraped with a 200 µL sterile pipette tip. After this, cells were twice washed with PBS and the culture medium containing LuzA or LuzD at concentrations of 10, 20, and 40 µM was added for an additional 48 h. The wound area was inspected at different times (0 h and 48 h) with the microscope Motic AE 20 and NIH Image software. (**B**) The percentage of cell migration was determined by measuring the width of the wound compared with the control. All experiments were repeated three times for each group (*n* = 18 for the control and each compound,*n*—quantity of photos). Results are expressed as the means ± standard deviation (SD). The asterisk (*) indicates a significant decrease in the migration of cells treated with luzonicosides compared with the non-treated cells (** *p* < 0.01, *** *p* < 0.001).

Metastatic spread is a main cause of death due to cancer. Consequently, the search for drugs which suppress the migration of cells, and thus metastasis formation, is a significant task for cancer research. Thus, we investigated the effect of LuzA and LuzD on cancer cell migration. Cancer cell migration and intravasation into blood/lymphatic vessels are crucial processes in metastatic spread [28]. LuzA inhibited the migration of highly-metastatic SK-Mel-28 cells by 71%, 74% or 97% at concentrations of 10, 20 or 40 µM, respectively, compared to non-treated cells (control) after 48 h of incubation. Interestingly, a similar activity was found for LuzD, with an inhibition of cell migration by 70%, 79%, and 95% compared with the control at the same concentrations (Figure 5). The migration process of cancer cells is thought to be regulated by specific molecules (receptors, kinases, proteases, etc.) included in the different signaling pathways. For instance, matrix metalloproteinases (MMPs) degraded the basement membrane, while protein kinases belonging to the PI3K/AKT pathway are associated with tumor cell invasion and metastasis [29,30]. The significant effects of LuzA and LuzD on the migration of SK-Mel-28 cells can be explained by the fact that these glycosides are likely to interact with specific targets which are highly expressed during the migration of cancer cells. However, to prove this suggestion, further investigation in this direction is needed.

## 3. Materials and Methods

### 3.1. Reagents and Drugs

The phosphate buffer saline (PBS), fetal bovine serum (FBS), Dulbecco’s Modified Eagle’s medium (DMEM), trypsin, l-glutamine, ethylenediaminetetraacetic acid (EDTA), agar, and gentamicin were purchased from Biolot (Moscow, Russia); anisomycin was purchased from NeoCorp (Weilheim, Germany); the CellTiter 96 nonradioactive cell proliferation assay kit was purchased from Promega (Madison, WI, USA).

### 3.2. Cell Culture

The SK-Mel-28 (ATCC # HTB-72^TM^) and RPMI-7951 (ATCC # HTB-66^TM^) melanoma cell lines were cultured as monolayer in DMEM medium, supplemented with 10% (*v*/*v*) heat-inactivated FBS, 2 mM l-glutamine, and 1% penicillin–streptomycin in a humidified atmosphere containing 5% CO_2_.

### 3.3. Cytotoxicity Assay

The cytotoxic activity of LuzA and LuzD was determined as previously described with slight modifications [31]. In brief, the SK-Mel-28 and RPMI-7951 cells were seeded (1 × 10^4^ cells/well) in 96-well plates in 200 µL of DMEM/10% FBS medium at 37 °C in a 5% CO_2_ incubator. After 24 h, the cells were treated with LuzA and LuzD in various concentrations (up to 200 µМ) for an additional 24 h at 37 °C in a 5% CO_2_ incubator. After incubation, 3-(4,5-dimethylthiazol-2-yl)-5-(3-carboxymethoxyphenyl)-2-(4-sulfophenyl)-2H-tetrazolium and inner salt (MTS reagent) (10 µL/well) was added and cells were then incubated for 4 h at 37 °C in a 5% CO_2_ atmosphere. Absorbance was measured at 490/630 nm.

### 3.4. Cell Proliferation Assay

The anti-proliferative activity of LuzA and LuzD was determined as previously described with slight modifications [32]. In brief, the SK-Mel-28 and RPMI-7951 (1 × 10^4^) cells were seeded in 96-well plates in 200 mL of DMEM medium at 37 °C in a 5% CO_2_ incubator. After 24 h, the medium was removed and replaced by a fresh medium containing 10, 20, or 40 µM of LuzA and LuzD. After incubation for 24, 48, or 72 h at 37 °C in a 5% CO_2_ atmosphere, MTS reagent (10 µL/well) was added and cells were then incubated for an additional 4 h at 37 °C in a 5% CO_2_ atmosphere. Absorbance was measured at 490/630 nm.

### 3.5. Cell Cycle Analysis and Detection of Apoptotic Cells

The cell cycle distribution was analyzed by flow cytometry using PI staining as described previously [33]. In brief, SK-Mel-28 cells were incubated overnight in 6-well plates (2 × 10^5^ cells/well). The medium was replaced with 1 mL of fresh media containing different concentrations of the substances. After 48 h of incubation, cells were harvested with a trypsin-EDTA solution, fixed with 70% EtOH/H_2_O, stained with PI/RNase buffer, and analysed by FACS. The results were quantitatively analyzed by Cell Quest Pro software (BD Bioscience, San Jose, CA, USA). The amount of the apoptotic cells was detected as a sub-G1 population [34]. Cells treated with anisomycin (aniso) (2 µM) were used as a positive control.

### 3.6. Western Blot Analysis

The extraction of proteins and the Western blot were done as described previously with modifications [35]. SK-Mel-28 cells were seeded in dishes (60 mm, 5 mL/dish, 1 × 10^6^ cells/dish). After 24 h, the cells were treated with a medium containing diffеrent concentrations of the glycosides. After 48 h, the cells were harvested and the proteins were extracted and subjected to electrophoresis in 12.5% SDS-polyacrylamide gels. Then, proteins were transferred to a polyvinylidene difluoride (PVDF) membrane. All procedures for the blocking of the membrane and developing the proteins were done as described in the manufacturer’s protocol. We detected signals with the enhanced chemiluminescence (ECL) system (Thermo Scientific, Rockford, IL, USA). Cells treated with anisomycin (2 µM) were used as a positive control.

### 3.7. Soft Agar Clonogenic Assay

A soft agar assay was performed on the SK-Mel-28 and RPMI-7951 cells as described earlier [31]. In brief, the cells (8 × 10^3^) were grown in 1 mL of 0.3% basal medium of Eagle’s agar containing 10% FBS. The cells were treated with LuzA and LuzD (10, 20, and 40 µM) and kept at 37 °C in a 5% CO_2_ atmosphere for 3 weeks. The colonies of SK-Mel-28 cells were scored using the microscope Motic AE 20 (Chengdu, China) and the Image J computer software program as described previously [36].

### 3.8. In Vitro Scratch Assay

The anti-metastatic activity of LuzA and LuzD was determined in accordance with our previous work [37]. SK-Mel-28 cells (3.0 × 10^5^ cells/mL) were seeded in 6-well plates. When cells were grown to an 80% confluence, the medium was removed and a wound was scraped with a 200 µL sterile pipette tip. After this, cells were twice washed with PBS and the culture medium containing LuzA or LuzD at concentrations of 10, 20, and 40 µM was added for an additional 48 h. The described experiments were repeated three times for each group. The wound area was inspected at different times (0 h and 48 h) with the microscope Motic AE 20 and NIH Image software (Image J). The width of the wound was measured and then the migration distance in cells was estimated as a percentage of each control for the wound closure area.

### 3.9. Data Analysis

All figures shown in this manuscript are representative of at least three independent experiments with similar results. Statistical analyses were performed using GraphPad Prism software v. 5.01 (GraphPad Prism software Inc., La Jolla, CA, USA). Data are presented as means ± SD (standard deviation). The unpaired Student’s *t*-test was used for the comparison of the two groups. Differences were considered to be statistically significant if *p* < 0.05.

## 4. Conclusions

In conclusion, the inhibitory activity of two cyclic steroid glycosides from the starfish *Echinaster luzonicus—*luzonicosides A and D—were investigated in human malignant melanoma cells. We have provided the evidence that both of the investigated glycosides suppressed the proliferation, colony formation, and migration of melanoma cells in vitro; however, luzonicoside A was more effective than luzonicoside D in these assays. The induction of apoptosis and G2/M cell cycle arrest were identified as potential mechanisms of action and were related to the regulation of cleaved caspase-3 and PARP, as well as Survivin, Bcl-2, p21 and Cyclin D1 expression levels. Overall, our results support the potential inhibitory efficacy of luzonicosides A and D on human malignant melanoma cells.
